# Redox Proteomics and Platelet Activation: Understanding the Redox Proteome to Improve Platelet Quality for Transfusion

**DOI:** 10.3390/ijms18020387

**Published:** 2017-02-11

**Authors:** Giona Sonego, Mélanie Abonnenc, Jean-Daniel Tissot, Michel Prudent, Niels Lion

**Affiliations:** 1 Transfusion Interrégionale Croix Rouge Suisse SA, Laboratoire de Recherche sur les Produits Sanguins, Route de la Corniche 2, 1066 Epalinges, Switzerland; giona.sonego@itransfusion.ch (G.S.); melanie.abonnenc@itransfusion.ch (M.A.); jean-daniel.tissot@itransfusion.ch (J.-D.T.); michel.prudent@itransfusion.ch (M.P.); 2 Faculté de Biologie et de Médecine, Université de Lausanne, CH-1011 Lausanne, Switzerland

**Keywords:** activation, aggregation, blood products, phosphorylation, platelet function, platelets, reactive oxygen species, redox cysteine, transfusion

## Abstract

Blood banks use pathogen inactivation (PI) technologies to increase the safety of platelet concentrates (PCs). The characteristics of PI-treated PCs slightly differ from those of untreated PCs, but the underlying reasons are not well understood. One possible cause is the generation of oxidative stress during the PI process. This is of great interest since reactive oxygen species (ROS) act as second messengers in platelet functions. Furthermore, there are links between protein oxidation and phosphorylation, another mechanism that is critical for cell regulation. Current research efforts focus on understanding the underlying mechanisms and identifying new target proteins. Proteomics technologies represent powerful tools for investigating signaling pathways involving ROS and post-translational modifications such as phosphorylation, while quantitative techniques enable the comparison of the platelet resting state versus the stimulated state. In particular, redox cysteine is a key player in platelet activation upon stimulation by different agonists. This review highlights the experiments that have provided insights into the roles of ROS in platelet function and the implications for platelet transfusion, and potentially in diseases such as inflammation and platelet hyperactivity. The review also describes the implication of redox mechanism in platelet storage considerations.

## 1. Introduction

**Figure 1:**
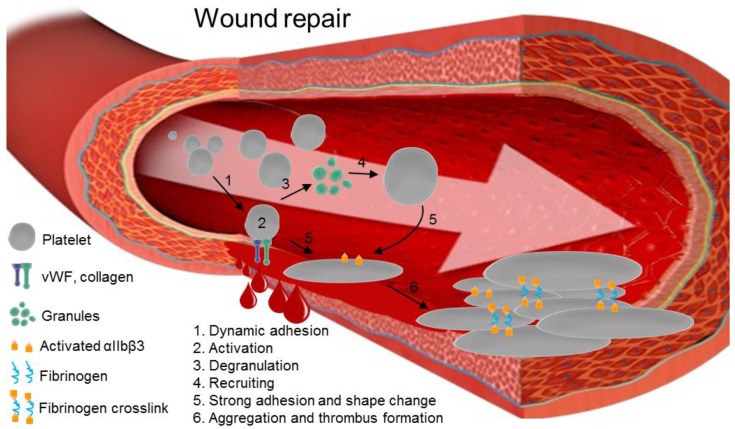
The steps in platelet activation and aggregation in thrombus formation after wounding. Damage to the epithelial wall of a vein exposes vWF and collagen. Receptors on platelets, such as glycoprotein Ib-IX (GP-Ib-IX), αIIbβ3, and glycoprotein VI (GPVI), reversibly bind to newly-exposed proteins (**1**). This induces internal signaling, leading to platelet activation (**2**). Platelets undergo degranulation (**3**) and secrete chemokines, including ADP, serotonin, ions like Ca^2+^, and other molecules that recruit additional platelets (**4**). Once activated platelets are bound on the endothelial wall, they progressively and definitively stick on the vein, changing their shape (**5**). Activated platelets form crosslinks via fibrinogen bridges between αIIbβ3 proteins, forming the thrombus (**6**).

Platelets are anucleate cells that are involved not only in hemostasis but also in many inflammatory processes [1,2]. Platelets are critical in transfusion practices. In the context of transfusion, platelet concentrates (PCs) are labile blood products used either prophylactically to prevent bleeding or therapeutically to stop bleeding (Figure 1) [3]. Cold storage of platelets induces modifications in platelet membranes. Cold storage also results in novel structures that are related to the associations of specific sugar structures that lead to the elimination of platelet after transfusion within the Kupffer cells of the liver [4]. Thus, PCs are mainly stored under agitation at room temperature (RT) until use for a maximum of five or seven days, depending on institutional specifications. Because there is a risk of bacterial growth during RT storage, as well as the danger of other emerging (undetected) pathogens, several countries now use pathogen inactivation technologies (PITs) to ensure a safer product [5]. Indeed, since the introduction of PITs adverse transfusion reactions and transfusion-transmitted infections have decreased dramatically [6]. Currently, there are two main commercially available PITs: INTERCEPT™ (Cerus, Concord, MA, USA) and Mirasol^®^ (Terumo BCT, Lakewood, CO, USA). A third PIT, THERAFLEX UV (Macopharma, Tourcoing, France), is being tested in a clinical phase III study. PITs exploit the anucleate nature of platelets and irreversibly crosslink or damage genetic material, thereby inactivating bacteria, viruses, and parasites [6].

While PITs have proven their efficacy for reducing pathogens in PCs [7], in vitro assays show that PI-treated PCs have altered functionality and accelerated metabolism. Importantly, treated PCs exhibit a higher platelet pre-activation level and are less reactive to stimuli, thus showing lower efficacy than untreated PCs [8,9,10]. In addition, a recent study reported that microparticulation was significantly increased in treated PCs as they approached their expiration date [11]. At the clinical level, Kerckhoffs et al. observed that inactivated platelets show decreased clinical effectiveness based on corrected count increment (CCI) after 1 h [12,13]. The result is an increased need for PCs. Nevertheless, Corash et al. expressed reservations about the use of PI-treated PCs [14]. The Cazenave [15] and Lozano groups [16] found no significant differences in the clinical effectiveness of PI-treated versus untreated PCs. Differences in data collection methods make it difficult to compare studies, so the clinical relevance of PITs in platelet function remains unclear in terms of CCI and bleeding time [17,18]. To summarize, there is a general consensus that PITs have a negative impact on the in vitro characteristics of PCs, but the reasons are not completely known or understood.

Oxidative stress is one possible cause of the functional changes in platelets that are observed in vitro after PIT treatment [19,20]. Indeed, amotosalen and riboflavin, which are the photosensitizers in INTERCEPT™ and Mirasol^®^, respectively, can sensitize oxygen molecules via type I or II mechanisms following excitation by UV light and thereby generate reactive oxygen species (ROS), like ^1^O_2_, O_2_^−·^, H_2_O_2_, and OH [21,22]. Furthermore, UV light alone can induce the formation of ROS [23], showing that UV(B) itself can play an active role in platelet functionality in vitro [24]. As a consequence, over time, an ROS imbalance could potentially impact platelet function in both reversible and irreversible ways.

Several diseases that involve platelets are accompanied or characterized by increased levels of ROS in their early stages. For instance, atherosclerosis, which occludes coronary vessels, involves platelet adhesion, which is enhanced by redox signaling [25]. Immune thrombocytopenia, which leads to decreased platelet production and increased platelet clearance, is also related to oxidative stress [26]. Atherothrombosis is the main risk factor in type I and II diabetes due to hyperactive platelets, and enhanced intracellular production of ROS is among the markers of these diseases [27]. Nevertheless, for more than 20 years ROS have not only been shown to be deleterious, but also to actively regulate platelet signaling and activation [28]. The results from recent redox proteomic approaches, together with classical approaches, highlight the importance of a complex redox cellular system that can influence the resting platelet state [29]. PITs could interfere with this state due to their intrinsic nature.

PITs are expected to alter the resting oxidative state of platelets by increasing both oxidation and redox second messenger signaling, for example in apoptosis [30] and in the platelet activation/aggregation pathway [31,32]. To clarify this, the redox proteome in platelets must be better understood. Redox proteomics provide tools that can describe these phenomena and that can be used to monitor both reversible and irreversible modifications of the protein oxidation state that play roles in biological structures and cell functionality [33]. Accordingly, redox proteomics can help define a given cellular state, such as aging, activation, and the pre-apoptotic state, and provide a better understanding of platelet functionality. Moreover, clinicians lack insights into the in vivo behavior of treated platelets after transfusion; thus, in the age of precision medicine, a better characterization of PCs could improve transfusion results.

The aim of this review is to provide a state-of-the-art description of the role of ROS in platelets, with an emphasis on cysteine, which is a key amino acid in redox signaling. The review describes how PITs are thought to damage the platelet proteome based on mass spectrometry results. It also analyzes platelet signaling that involves ROS in order to better understand the impact of reversible oxidation. Within the context of transfusion, the review gives an overview of the cysteine redox state and its crosstalk with phosphorylation. Finally, discussions are opened on the actual debate on platelet processing/storage strategies.

## 2. Alterations and Oxidation of Platelet Proteins

Platelet storage lesions affect cell metabolism, activation and morphology [34,35,36]. Such lesions have a non-negligible impact on proteins [37]. Indeed, the use of a combination of multiple proteomic strategies has revealed a network of proteins involved in platelet activation, cytoskeletal reorganization, vesicle trafficking, and apoptosis, all of which undergo storage-related changes [37]. To limit storage-induced stress on platelets, efforts are being made to continuously optimize storage conditions, e.g., by making changes in the composition of plastic bags and additive solutions as well as by changing the temperature and agitation conditions. PITs have also been found to exacerbate platelet storage lesions [37]. Even though platelets are enucleated cells, the platelets remain capable of synthesizing some proteins [36,38,39,40]. Indeed, much attention has been focused on the platelet proteome, which involves more than 4000 proteins to date [41], in order to better define the impact of PITs on platelet function.

Marrocco et al. used a two dimensional-gel electrophoresis (2D-GE) approach to analyze changes in platelet proteins after riboflavin/UVB treatment. They found significant changes in the expression of some relevant proteins, including plekstrin, β-actin, ATP synthase, and the nuclear chloride channel [42]. Our laboratory used a similar approach and found significant changes in the expression of two proteins, DJ-1 and glutaredoxin-5, after platelets were subjected to INTERCEPT™-induced oxidative stress [43]. In addition, Hechler et al. identified three distinct protein pattern variations (involving plekstrin, integrin-linked protein kinase, and the cytoplasmic protein NCK2) in INTERCEPT™-treated platelets, also by using 2D-GE [44]. Thiele et al. used a gel-free approach in which liquid chromatography was coupled to tandem mass spectrometry to analyze buffy coat-derived PCs. They identified changes in a number of proteins after PI treatment. The most important and irreversible alterations, observed on days 1 and 5, were alterations in the expression levels of the PEAR-1, CXCR4, and CLIC4 proteins [45].

These changes in protein expression can probably be attributed, at least in part, to protein oxidation processes such as carbonylation. Supporting this notion, Johnson et al. found higher levels of carbonylation in Mirasol^®^-treated PCs compared to untreated buffy coat-derived PCs [20]. Carbonyl insertion into proteins occurs mainly by three mechanisms: amino acid backbone scission, amino acid side chain cleavage, and side chain ketone or aldehyde formation by metal catalysis [46]. Diverse amino acid side chains can undergo oxidation, with lysine, arginine, proline, threonine, and tryptophan among the most sensitive amino acids [47,48,49]. Oxygen insertion can drive an important electronic change and affect the amino acid structure. In addition, singlet oxygen can drive inter-amino acid reactions that induce the formation of covalent bridges and influence the tertiary structure of proteins [50].

Carbonylation constitutes an intracellular biomarker of oxidative stress that is often associated with aging and diseases. It is one of the most common types of protein damage, notably because it is an irreversible and stable modification that is usually not affected by sample preparation. During aging, antioxidant cell defenses decrease [51], allowing ROS to interact with DNA, RNA, lipids, and proteins. In the same way, diseases accompanied by oxidative stress raise the carbonylation level in the platelet proteome [52]. In general, carbonylated proteins are either repaired by specific enzymes or processed by the proteolysis machinery. These oxidation reactions can induce partial protein unfolding, thereby exposing hydrophobic regions and leading to the loss of the protein’s primary function and to aggregation [20,53]. However, while protein carbonylation is generally considered to be deleterious and associated with diseases [47], it might well be one of the final steps of the oxidation chain caused by ROS in platelet activation that participates in redox signaling [54].

Aging and disease might not be the only causes of protein carbonylation in cells. Upon platelet activation, the platelet proteome undergoes a profound transformation, and the level of protein carbonylation increases [55]. For instance, thrombin and arachidonic acid stimulation enhance the carbonylation of platelet proteins [56]. This suggests the involvement of ROS in platelet activation, leading to carbonylation. Although a few proteins show changes after PI treatment [45], the findings are difficult to interpret; the findings may partially explain the changes in platelet functionality that are observed in in vitro tests. In addition, the global impact of carbonylation on the proteome is relatively low. It is reasonable to hypothesize that subtle changes might occur during PIT treatment or even during platelet activation in vivo, such as regulation of the redox state.

## 3. The Role of ROS in Platelet Function

### 3.1. Platelet Activation Pathways Stimulate ROS Production

There are a few agonist-induced activation pathways that produce ROS, including the collagen, thrombin, and arachidonic acid activation pathways. The 2′,7′*-*dichlorodihydrofluorescein diacetate (H_2_DCFDA) assay is most often used to measure the ROS level inside cells. After cytosolic hydrolysis, H_2_DCFDA can be oxidized into 2′,7′*-*dichlorofluorescein (DCF), which fluoresces at 520 nm. H_2_DCFDA is currently used in flow cytometry analyses for direct and reliable relative quantification of intracellular ROS. After cytosolic hydrolysis, DCFH can be oxidized into DCF, which fluoresces at 520 nm. H_2_DCFDA is currently used in flow cytometry analyses for direct and reliable relative quantification of intracellular ROS. H_2_DCFDA is currently used in flow cytometry, allowing direct and reliable relative quantification of intracellular ROS. One of the main advantages of this technique is that it allows the rapid detection of ROS, so that short-lived oxidative imbalance can be measured. Using an H_2_DCFDA-based assay, Bakdash and Williams found compartmentalization of ROS production that depended on the stimulated receptor [57]. Stimulation of platelets with convulxin, an agonist peptide targeting the collagen glycoprotein VI (GPVI) receptor, induces intraplatelet ROS production, while thrombin, a GPIbα and PARs agonist, induces mainly extracellular ROS formation. Indeed, upon thrombin activation, Bakdash and William found significantly decreased P-selectin exposure and αIIbβ3 activation using a combination of the extracellular antioxidants superoxide dismutase (SOD) and catalase [57]. Carrim et al. showed that a concerted mechanism involving GPIbα and PAR-4 receptors, the latter of which has a low affinity for thrombin, is involved in generating thrombin-derived ROS [58]. Indeed, specific cleavage of the thrombin-binding site on GPIbα by the Nk protease at Tyr276–Asp277 abolishes ROS generation under thrombin stimulation. In addition, in the presence of the Nk protease, stimulation with PAR-4 agonists such as PAR4-AP and AYPGKF-NH_2_ substantially decreases the platelet aggregation percentage [58]. Upon collagen stimulation, ROS generation is significantly decreased by inhibiting focal adhesion tyrosine kinase in the GPIbα–PAR4 downstream cascade [59], while the main receptor, PAR-1, which has a high affinity for thrombin, seems to activate platelets via ROS-independent pathways. Recently, cyclophilin A (CyPA), a bidirectional regulator of the αIIbβ3 receptor, was identified as a crucial component in the generation of ROS under thrombin stimulation [60]. CyPA interacts with the p47^phox^ subunit to modulate the assembly of the cytoplasmic membrane NAD(P)H complex, which generates ROS [61]. Furthermore, flow cytometry shows that thrombin-induced ROS formation and platelet activation via αIIbβ3, which was quantified using an antibody to PAC-1 (the first procaspase activating compound), are significantly reduced in CyPA-deficient mice (CyPA^−/−^) [60].

Collagen is probably one of the most potent agonists that stimulate ROS-mediated platelet activation [62]. GPVI, a platelet-specific collagen receptor, has been identified as a receptor that regulates platelet redox [63]. Arthur et al. showed by immunoprecipitation that the ligation of the GPVI/FcRγ needs the TNF adapter receptor 4 (TRAF4) to act downstream to enhance ROS production by NADPH oxidase (NOX) [64]. Indeed, the bridging interaction of TRAF4 with the intracellular GPVI peptide and the p47^phox^ subunit of NOX allows transduction of the stimuli, which culminates in the formation of the thrombus. TRAF4 also interacts with other proteins, such as Hic-5, which is linked to the Src family tyrosine kinase Lyn, in collagen-mediated signal propagation [65]. Moreover, the same group identified two distinct phases of ROS production. ROS were measured at an early stage in the presence of a spleen tyrosine kinase (Syk) inhibitor, BAY61-3606. However, 15 to 20 min later, the second wave of ROS was not observed in the presence of the inhibitor. They concluded that collagen-mediated platelet activation involves two pathways, a Syk-independent pathway and a Syk-dependent pathway. This suggests a potential role for ROS in phosphorylation events (see Section 5).

At a later stage, during thrombus formation, oxidative stress leads to enhanced phospholipase A2 enzyme activity, resulting in the release of arachidonic acid into the cytoplasm [66]. Activated platelets trigger the conversion of arachidonic acid into a potent platelet aggregation mediator, thromboxane A_2_ (TXA_2_). TXA_2_ has a pivotal role in that it stabilizes the thrombus and promotes thrombus growth by recruiting additional platelets. During the conversion of arachidonic acid into TXA_2_, cyclooxigenase 1 (COX1) catalyzes the formation of the intermediate prostaglandin H_2_ and generates ROS as a by-product [67]. Interestingly, other agonists, such as ADP, soluble CD40 ligand, and other chemokines, do not detectably increase ROS [31]. This also indicates that ROS production occurs at a specific time and via a specific pathway during platelet activation.

### 3.2. NADPH Oxidase

**Figure 2:**
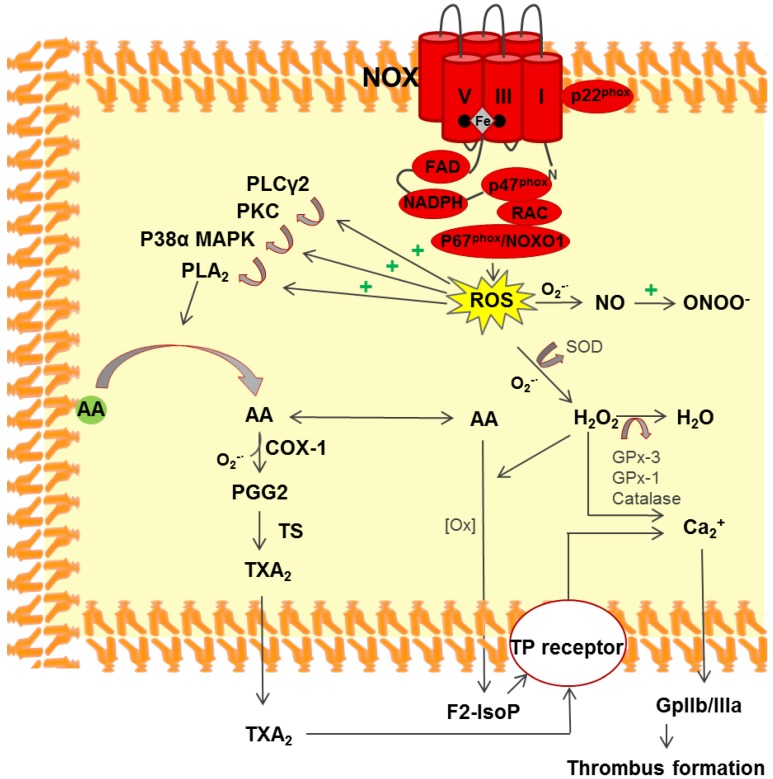
The role of NOX (NOX1 and/or NOX2) in thrombus formation via ROS production. Increased intracellular ROS production enhances (+) redox internal signaling. ROS potentiate the PLCγ/PKC/MAPKp38 signaling cascade, thereby inducing phospholipase A2 (PLA_2_) activation via (arrows) phosphorylation. PLA_2_ catalyzes the hydrolysis of the phospholipid sn2-acyl bond at the membrane, releasing arachidonic acid (AA) into the cytoplasm. AA can either be converted by COX1 into prostaglandin G2 (PGG2), an intermediate in the synthesis of thromboxane A2 (TxA_2_), or it can be oxidized by peroxides to form F2 isoprostane (F2-IsoP). Both can bind the prostanoid TP receptor during thrombosis/hemostasis. O_2_^−·^ can be converted into the more membrane permeable H_2_O_2_ by superoxide dismutase (SOD). H_2_O_2_ acts as a second messenger in various cellular signaling pathways, and it also induces Ca^2+^ mobilization. Ca^2+^ mobilization precede the glycoprotein GPIIb/IIIa (or αIIbβ3) affinity modulation to fibrinogen binding. Alternatively, O_2_^−·^ reacts with NO, leading to reactive nitrogen species such as ONOO^−^.

The role of ROS as messengers or mediators of cell signaling is currently widely accepted [68]. Several platelet-related pathways that produce and release ROS have been identified, including the NOX, COX1, and xanthine pathways [69]. Whereas the latter two pathways produce ROS as a by-product, the primary function of the NOX pathway is to modulate redox-sensitive signaling by converting molecular oxygen into the oxygen superoxide anion, O_2_^−·^ (Figure 2). This involves the inactivation of phosphotyrosine phosphatases, the activation of certain transcription factors, and the modulation of ion channels [70]. The NOX pathway not only produces ROS, but it can also be activated by ROS. Indeed, one hallmark of this pathway is that it responds to a large range of stimuli, including chemical, physical, environmental, and biological factors [71]. The NOX complex is activated by cytoplasmic p47^phox^ phosphorylation, which causes it to localize to the cell membrane. Finazzi-Agrò et al. found that the NOX complex is an essential component for efficient activation in platelets, and they demonstrated the release of H_2_O_2_ upon platelet activation using scopoletin in a fluorescence assay [72]. NOX-derived ROS participate in the core stress-response signaling pathway via the MAPK and c-Jun NH_2_-terminal kinases [71]. Several groups have investigated the role of ROS production during platelet activation using NOX^−/−^ mouse cells. Recently, Delaney et al. showed that enzymatic NOX1 and NOX2 complexes are not part of the same platelet activation pathway [73]. Walsh et al. demonstrated the role of NOX1 in optimal p38MAPK signaling and in subsequent TXA_2_ production in C57BL6/J background mice [74]. Inhibition of NOX1, either by chemicals such as 2-acetylphenothiazine, diphenylene iodonium, or apocynin, or by the specific peptide-antagonist gp91, decreases calcium mobilization, which is associated with a decrease in platelet αIIbβ3 activation, aggregation, and thrombus formation under high shear flow conditions [31,74,75]. On the other hand, NOX2 knock-out potently inhibits ROS generation under collagen peptide stimulation conditions [73]. In addition, performing the laser-induced arterial thrombosis assay in NOX2^−/−^ mice showed an effect on thrombus formation but not on bleeding time [73]. These data suggest that ROS are crucial for efficient hemostasis, while platelet adhesion is less ROS-dependent. In the same way, ROS scavengers are able to reduce the platelet response to stimuli, highlighting the importance of ROS as secondary messengers [31,76].

### 3.3. Platelet Activation via ROS

Platelet activation is a complex biological process that involves several signaling pathways [77,78]. Early work in this field established that platelet activation occurs via ROS stimulation, but the underlying regulatory mechanisms and the target molecules were not well known. The O_2_^−·^ generated by NOX can be converted by SOD to H_2_O_2_, which is more stable and acts as a second messenger, or to a hydroxyl radical (OH) [28]. O_2_^−·^ can also stimulate F2-isoprostane and interact with nitric oxide, which downregulates platelet activation to form ONOO^−^ (Figure 2). To confirm the role of H_2_O_2_ in platelet activation, Dayal et al. overexpressed glutathione-peroxidase-1 in mice [79] and found significantly reduced platelet activation in response to thrombin stimulation due to the scavenging of H_2_O_2_ by this peroxidase.

**Figure 3:**
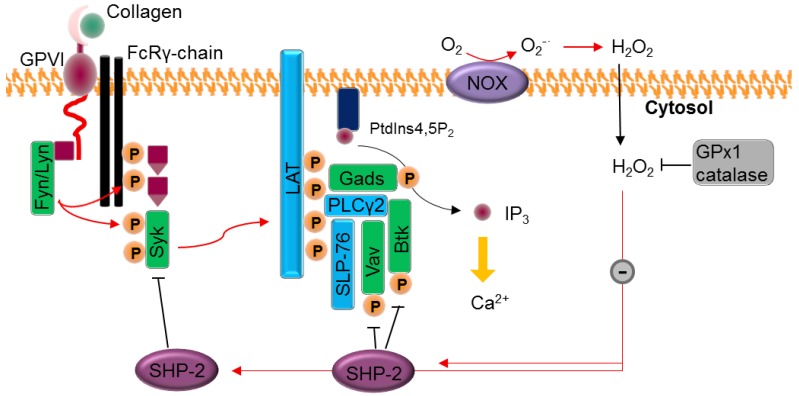
Ca^2+^ mobilization upon stimulation with collagen agonists. Collagen induces the assembly of NOX complexes at the membrane. O_2_^−·^ is converted by SOD into the thiol effector and membrane permeable (black arrow) H_2_O_2_, which reversibly switches off the SHP-2 phosphatase SHP-2 cysteine oxidation inhibits its phosphatase function (black T bars) and results in enhanced (red arrows) kinases Syk, Vav1, and Btk phosphorylation, essential for the activation of membrane complexes such as PLCγ-2 and LAT and inducing IP_3_ release in the cytosol. IP_3_ acts on the calcium channel, mobilizing Ca^2+^ (yellow arrow). Adapted from reference [31], courtesy of Tong-Shin Chang.

Apparently intra-platelet ROS generation is related to αIIbβ3 regulation, rather than to granule secretion or changes in the shape of platelets [31]. The work of Jang et al. has provided insights into the collagen/platelet aggregation pathway (Figure 3) [80]. After stimulation of GPVI with collagen, the Src kinase family of proteins initiates a signaling cascade by phosphorylating the Fc-receptor γ-chain, which recruits the tyrosine kinase Syk. Syk activates the linker of activated T cells, which induces the formation of a signaling complex with phospholipase Cγ (PLCγ-2). The subsequent phosphorylation of PLCγ-2 triggers the hydrolysis of the membrane phospholipid PIP_2_ into soluble inositol trisphosphate (IP_3_) and other signaling molecules. Lastly, once it is bound to the IP_3_ receptor (InsP3R, a membrane glycoprotein that acts as a calcium channel), IP_3_ induces calcium ion mobilization in the cytosol. Jang et al. identified a redox cysteine switch-off in SHP-2 that plays an important role in regulating the tyrosine kinases that target the Syk and PLCγ-2 complex [80]. They indirectly measured cysteine sulfenylation (i.e., R-SOH) by ligation with thiol-reactive biotin to confirm the inhibitory effect of H_2_O_2_ on SHP-2. Furthermore, Krötz et al. showed that the addition of SOD to collagen-stimulated platelets reduced the release of ADP into the supernatant, thus decreasing platelet recruitment [32]. This could indicate that O_2_^−·^ participates in platelet recruitment, while H_2_O_2_ (formed by the reduction of O_2_^−·^ by SOD) is not involved. Taken together, these studies strongly suggest that ROS partially regulate the activation of integrin αIIbβ3 and the subsequent aggregation of platelets and thrombus growth (Figure 1).

ROS second messengers also negatively regulate platelet function by inducing the shedding of the platelet GPIbα ectodomain and thus reducing vWF binding [81]. When platelets that were previously incubated with antioxidants such as *N*-acetyl cysteine or DTT are stimulated with collagen, thrombin or A23187, shedding of the ectodomain is partially inhibited. ADAM17/TACE enzyme activation, which occurs via a p38MAPK-dependent mechanism that is related to intraplatelet oxidative stress, enables the release of the GPIbα ectodomain [82]. Strong stimulation of platelets and ROS enhance the formation of the mitochondrial permeability transition (mPT) pore, allowing cytoplasmic calcium to enter mitochondria. In turn, this stimulates agonist-initiated phosphatidylserine exposure at the membrane [83]. It appears that thiol groups that are able to cross-link play a critical role in mPT pore formation, explaining the involvement of ROS. Indeed, McStay et al. showed that under conditions of oxidative stress, adenine nucleotide translocase can form an intramolecular cross-link between C160 and C257, blocking ATP/ADP exchange at the mitochondrial membrane and enhancing the formation of the mPT pore [84]. In the context of transfusion medicine, PCs subjected to Mirasol^®^ treatment showed increased ROS production during storage and specifically on days 2 and 5 of storage [20]. These findings may explain the activation state of inactivated platelets: the redox signaling pathways could be overstimulated due to the positive ROS imbalance [51] relative to the resting state.

## 4. Role of Redox Cysteine in Platelets

Free thiol groups and thiol–disulfide exchanges play central roles in the responses of platelets to stimuli [85]. For example, the reduction of a functional disulfide bond in the β3 extracellular subunit of the αIIbβ3 receptor, which possesses a redox switch, via inside-out signaling induces conformational changes that enhance platelet activation [86]. PI-treated PCs, in particular UVB-treated platelets, have an increased affinity to the PAC-1 antibody, suggesting that the αIIbβ3 receptor undergoes a conformational change into its active form [8]. In the same way, excessive exposure to UVC illumination disrupts the disulfide bond in αIIbβ3, thereby enhancing the affinity of the Arg–Gly–Asp (or RGD) motif for fibrinogen [87]. These studies suggest that UV light has a non-negligible impact on the redox system in platelets.

Free cysteine residues are the preferred targets for oxidation reactions, and they could be involved in sensing cellular oxidative stress, as part of a defense mechanism or in cellular regulation. Since the thiolate (R–S^−^) attacks the H_2_O_2_, the redox potential of protein-free thiols is influenced mainly by the chemical environment and can modulate R–SH p*K*_a_. The reactivity of protein-free thiols could depend on their accessibility and steric hindrance, since thiol oxidations are substitution nucleophile bi-molecular (SN2) reactions, implying a concerted backbone in the intermediate [88]. Accordingly, bulky groups surrounding an R–SH group will decrease its reactivity. Importantly, thiols are good oxygen acceptors and, among other oxidation states not listed here, can be reversibly transformed into R–S–S–R, R–SOH or R–SO_2_H or irreversibly transformed into RSO_3_H (Table 1). Here we only discuss reversible cysteine oxidation. Indeed, reversible cysteine oxidation is at the core of the second H_2_O_2_ messenger system and, to the best of our knowledge, has not been exhaustively studied in platelets. Posttranslational cysteine oxidation is reviewed elsewhere [89].

### 4.1. The Involvement of Cysteine in ROS Sensing and Defense

Oxidative stress plays an active role in programmed cell death. Farah and Amberg hypothesized that the oxidation of cysteine residues in actin is a conserved cellular mechanism that senses oxidative stress and accelerates cell death [90]. This hypothesis was based on their study in yeast, where the oxidation of the highly conserved actin Cys-285 and Cys-374 residues impacts actin filaments (F-actin) polymerization and increases the ROS level. Fixation of the polymerization state of cytoskeletal actin is part of the apoptosis pathway. Actin assembly and disassembly are not only important in apoptosis; they are also related to changes in the shapes and the rapid spread of platelets during adhesion hemostasis (see Section 4.2).

The in vitro hypotonic shock response assay, which tests the capacity of platelets to recover their initial shape after hypotonic stimulation, showed different results for control platelets versus PI-treated platelets [24,91,92]. It would be interesting to verify the correlation between ROS imbalance and poorer cell shape recovery. Notably, PCs have a consistent plasma fraction that contains albumin, the most abundant plasma protein. Albumin is an important regulator of blood osmotic pressure, and it transports several substances, like hormones, in the blood. Albumin has also been proposed to act as an antioxidant, thanks to its high concentration and its ability to capture ROS with its free cysteine (Cys34). Carbalal et al. proposed sulfenylation of human serum albumin as a mechanism that could protect plasma free thiols via a mechanism in which ROS are stabilized by Cys34 before the formation of a disulfide bond [93]. This point of view considers R–SOH to be an intermediate in the formation of the R–S–S–G moiety, protecting cysteine from further oxidation [89]. As a consequence, albumin could counter increases in ROS in PCs, thereby playing a protective role against platelet lesions.

In the cytosol, the DJ-1 protein (Parkinson disease protein 7 or PARK7) is suspected to be an oxidative stress sensor because of its highly conserved Cys106 residue, and it may help protect the mitochondrial membrane [43]. In our laboratory, we used 2D-GE to show a pI shift of the DJ-1 spot after INTERCEPT™ treatment, suggesting the presence of an oxidized form of the protein [43]. After this treatment, more than 20% of the DJ-1 protein was in its oxidized form in PCs [43].

Peroxiredoxin-2 (PrxII) has a similar protective effect against undesired platelet activation and thrombosis. Recently, the antioxidant behavior of PrxII was correlated with platelet activation by Jang’s research group, who published a study on SHP-2 [94]. Indeed, PrxII downregulates GPVI-mediated platelet activation by efficiently trapping H_2_O_2_, thereby preventing SHP-2 sulfenylation and subsequent inactivation. PrxII antithrombotic activity was also validated in vivo using an arterial injury assay to investigate mice lacking the *PRDXII* gene [94].

### 4.2. Cysteine-Mediated Redox Signaling

Cells can exploit reversible thiol oxidation to transduce stimuli, for example via disulfide bonds and sulfenic acids. The difficulties in detecting the sulfenic acid moiety, –SOH, is due mainly to its much lower p*K*_a_: it easily undergoes RSO^−^, which is a better nucleophile then thiolate R–S^−^, and this enhances its reactivity with other molecules [95]. This reversible and therefore transitory oxidation-based post-translational modification (oxPTM) is probably, like phosphorylation-based PTMs, part of a binary switch that modulates protein activity [96]. Oxidative disulfide formation is enhanced by H_2_O_2_ and can be involved in the formation of protein complexes or conformational changes that modulate protein function. Phosphotyrosine phosphatases are among the best understood examples of oxidative disulfide formation. In this case, a free cysteine thiol is reversibly converted to a sulfenic acid, inactivating the phosphatase function. It has been proposed that such oxPTMs are part of a widespread and highly conserved redox regulation mechanism that works in conjunction with receptor tyrosine kinases [97]. Inhibition of phosphotyrosine phosphatases enhances tyrosine phosphorylation (e.g., SHP2; see Section 3.3). There are other sulfenic acid-mediated mechanisms as well. In addition to oxidative folding, which occurs mainly in the endoplasmic reticulum, disulfide bond formation that is driven by R–SOH intermediates is involved in the assembly of protein complexes. One example is protein kinase A, in which disulfide bond formation between regulatory subunits is driven by an R–SOH intermediate and acts to induce the translocation of a protein complex from the cytosol to the cytoplasmic membrane [98].

Disulfide bonds should not only be considered entities that stabilize protein tertiary structure or that act as a defense mechanism. Indeed, Fiaschi et al. showed the importance of the formation of a mixed disulfide bond (i.e., the glutathionylation of actin Cys374) in enhancing the dynamic spread of murine fibroblasts and their adhesion via cytoskeletal rearrangement [99]. Their conclusion was based on the observation that glutathione depletion or the Cys–374–Ala substitution importantly affected actin fiber formation and fibroblast adhesion, further confirming their model based on the involvement of ROS production on integrin-mediated adhesion to extracellular matrix [100,101]. Thiol-disulfide exchanges also represent a redox switch in protein function [102]. The thiol oxidoreductase protein disulfide isomerase (PDI) is particularly interesting in the context of this review because it is secreted (inside T-granules) by platelets during activation [103]. Platelet PDI targets the extracellular domain of αIIbβ3 and therefore mediates thrombus formation. Indeed, intravital microscopy demonstrated that the accumulation of PDI at the site of laser-induced arteriolar wall injury was markedly decreased in β3^−/−^ mice [104]. Another mechanism by which PDI participates in thrombus formation involves the upregulation of NOX1 and NOX2, which increases ROS production [105].

Several PC proteins that are affected by PITs are mitochondrial proteins or proteins that are involved in preserving mitochondrial integrity [37]. The mitochondrial environment is the richest environment in the cell in terms of free cysteine thiols, and this contributes to mitochondrial characteristics and function [88]. Given the alkaline environment of the inner mitochondria due to protons being pumped into the intermembrane space, free cysteines are likely to be in the thiolate form. ROS are produced continuously along the oxidative phosphorylation chain. As a consequence, free thiols can easily be oxidized to R–SOH or even to higher oxidation states (Table 1). In the mitochondrial matrix, as in other cell compartments, glutathione reacts with thiolates to protect protein function when there is an H_2_O_2_ imbalance.

## 5. ROS-Phosphorylation Crosstalk

Protein phosphorylation is one of the most important regulatory mechanisms in cell biology [106]. Among the reversible PTMs, phosphorylation is one of the most studied and best understood. The types and levels of kinases and phosphatases inside cells highlight the importance of phosphorylation as a regulatory mechanism. Furthermore, kinases and phosphatases are often in competition with respect to their target proteins, and many proteins and enzymes are regulated by phosphorylation at multiple sites. Protein phosphorylation is easily detected by Western blot analysis and by 2D-GE techniques [107]. Current mass spectrometry-based proteomic techniques permit the study of the phosphorylation level after phospho-peptide enrichment, allowing researchers to conduct untargeted experiments and thereby gain an overview of phosphorylation in cells [108,109]. In signal transduction, phosphorylation is often induced by first messengers, such as hormones and paracrine/autocrine agents, and it can act in a concerted way with second messengers like calcium ions, redox signaling molecules, nitric oxide and lipophilic molecules.

Interestingly, the link between UVB-ROS-phosphorylation and platelet activation was suggested by Van Marwijk Kooy et al. in 1993 [110]. They observed that after UVB illumination of platelets, there was increased fibrinogen binding that correlated with a 40% higher level of phosphorylation downstream of protein kinase C. UV-induced phosphorylation and platelet aggregation were significantly reduced in the presence of SOD and catalase, respectively, which are scavengers of O_2_^−·^ and H_2_O_2_. They concluded that the ROS generated upon UVB exposure induces platelet activation-aggregation via protein kinase C. Later, Schubert’s group provided an important example of the impact of UVB-based PIT on the PTM proteome [111]. They used 1D gel semi-quantitative proteomics and phospho-immunoblotting and found that apheresis platelet bags resulted in enhanced phosphorylation of the vasodilator-stimulated phosphoprotein (VASP) after riboflavin-UVB treatment [111]. VASP is a highly concentrated cytosolic platelet protein involved in the ADP activation cascade, which promotes F-actin elongation. Its phosphorylation on Ser-157 and Ser-239 strongly correlates with αIIbβ3 binding to soluble fibrinogen and with platelet aggregation [112]. This observation led to the development of an in vitro assay that is currently in clinical use that tests the efficacy of antiplatelet treatments (such as clopidogrel, which binds to the P2Y12 receptor) on VASP phosphorylation [113]. This group investigated upstream kinase activity and showed that inhibiting p38MAPK after PIT treatment (Mirasol^®^ in their study) significantly restored PC quality based on in vitro tests [114]. Indeed, the phosphorylation level was tested in treated versus untreated PCs, and the results showed that kinases had enhanced activity after exposure to UVB. In particular, p38MAPK showed a greater than two-fold increase in activity [114]. Finally, they demonstrated that the enhanced p38MAPK kinase activity induced by Mirasol^®^ treatment triggered apoptosis in apheresis PCs [115]. Moreover, they also showed that Mirasol^®^ treatment potentiates the phosphorylation of NF-κB and IκBα, enhancing redox-sensitive transcription activity [20]. In the context of this review, these findings suggest that the ROS produced during pathogen inactivation can interfere with protein phosphorylation and impact internal cell signaling.

The possible links between oxidative stress and cell signaling raise some fascinating possibilities. Recently, in human epidermoid carcinoma cells, Carroll’s group demonstrated that endothelial growth factor receptor (EGFR) kinase activity is modulated by the oxidation of Cys979 into sulfenic acid after H_2_O_2_ reacts with its free thiol [116]. They optimized an experimental strategy for the site-specific analysis of protein –SOH that uses a sulfenic acid-specific probe (DYn-2) to fix this labile redox cysteine state. This allows sulfenylation to be studied in cells over time, and they specifically compared the basal sulfenylome with one in stressed conditions following H_2_O_2_ stimulation of RKO and A431 cells [117]. The identification of the sulfenic acid sites allowed the mechanistic study of activation-translocation of the transcription factor HIF1A by SIRT6. Our laboratory is currently using this strategy to characterize the platelet sulfenylome (Sonego, G.; Prudent, M.; et al., Mapping the platelet sulfenylome, Manuscript in writing process). Our aim is to assess the impact of PITs on PCs and to elucidate the connection between ROS second messengers that are produced during PC treatment and PC functionality in vitro.

## 6. The Overlap of Redox Proteomics and Transfusion

The storage conditions for PCs are continuously being changed and improved, with the result that different countries use different storage conditions. These changes also raise the issue of the cost-effectiveness of incremental improvements. Changes in storage conditions have suggested interesting new possibilities, and clinical trials must be continuously updated to reflect the changes. Moreover, improved clinical monitoring methods and the use of advanced analytical tests are being used to reassess abandoned practices for use in specific applications [118]. For example, whole blood storage, platelet cryopreservation and cold storage have been reconsidered for hemorrhagic shock treatment [119,120,121]. Cold storage at 4 °C has shown positive outcomes, and platelet function is conserved until day 15 in these conditions [119]. Therefore the field is evolving, and there is a danger that debate about the convenience of RT platelet storage could destabilize the paradigm. Along with other authors, Cap has criticized the RT storage, asserting that post-transfusion platelet recovery is not a universal criterion for platelet function and does not guarantee efficacy in the context of the targeted condition beyond prophylactic treatment [122]. While the debate over optimal PC storage conditions is not the focus of the present review, in terms of the advantages and disadvantages, costs, product availability, effectiveness, etc., we would like to make the point that “omic” research approaches can help clinicians and blood bankers make better medical decisions.

### 6.1. The Impact of Antioxidants on PI-Treated PCs

PITs are photochemical treatments or photo-treatments that induce oxidative lesions. Our lab has demonstrated the effects of PITs on oxidative processes in platelets by showing an important decrease in extracellular antioxidant power after INTERCEPT^®^ treatment; the same effects were seen with our laboratory’s in-house riboflavin/UVB system [24]. Antioxidant power was measured electrochemically using EDEL technology (in arbitrary units equivalent to one micromolar ascorbic acid) by deposing a 2 µL drop of liquid sample on a microchip. [123,124]. Using the results, our group developed a quality control assay to assess the effects of PIT before product delivery [123]. One solution for countering the oxidative damage of PITs that has been proposed in the literature is to supplement PCs with antioxidants. A few studies have tested the ability of added antioxidants to decrease the carbonylation level during platelet storage/aging and to reduce oxidative stress [125,126]. The idea is that antioxidants will directly scavenge ROS and protect cellular components like glutathione. However, this supplementation is questionable and would have to be fine-tuned as antioxidants clearly modulate platelet function.

Olas and Wachowitz studied the effect of resveratrol and vitamin C on the inhibition of ROS production [127]. Vitamin C concentrations of 0.75 to 3 mM showed good effectiveness against ROS, while resveratrol only partially inhibited ROS production. Sobotkova et al. showed the antioxidant properties of resveratrol on washed platelets using trolox, a hydrophilic analog of vitamin E, using an equivalent antioxidant capacity assay and indirect measurements of OH· following salicylic acid hydroxylation into 2,3-DHB and 2,5-DHB [128]. Importantly, resveratrol, which is naturally found in wine, seems to modulate key proteins involved in intracellular signaling, including effects on protein kinase activation (p38MAPK), phospholipase C, the Ca^2+^ cascade and the activation of NO/cyclic GMP [129]. However, due to suggested differences in redox-sensitive pathways, the inhibition of platelet activation by antioxidants remains limited by their specific properties [57]. ROS production that is induced by PIT may be randomly distributed inside the cell or it may be concentrated in the vicinity of DNA and RNA molecules, especially in the case of amotosalen, which is an intercalation compound with chemical activity that differs from riboflavin. In addition, there is a risk that the use of antioxidants during the PI process will decrease the PI efficacy by quenching the effect of the treatment. Lastly, antioxidants such as vitamin E and β-carotene inhibit the primary function of platelets and are suspected of being associated with hemorrhagic stroke in male patients with lung cancer [130]. Antioxidants that protect supernatant proteins and the integrity of platelet membranes probably have the best chance of success.

### 6.2. Cold Storage and Cryopreservation

There are several possible strategies for reducing or eliminating the risk of bacterial growth during RT storage while avoiding the use of PITs and without shortening storage time. Cold storage and cryopreservation are among the possible solutions. Cold storage has not been in general use since the 1970s. Despite shortened recirculation issues, recent clinical studies have shown that cold-stored products have potential for use for specific treatments [131]. Cryopreserved platelets have an obvious advantage in terms of overcoming the issue of short shelf life. Recently, autologous cryopreserved platelets were successfully transfused into HLA-alloimmunized patients, showing good conservation of their function in the presence of strong agonists [132]. However, relative to storage at RT, low temperature storage (both at 4 and −80 °C) results in platelet hyperactivity. In cryopreserved platelets, it has been hypothesized that the development of a pro-coagulant phenotype at the membrane underlies the altered in vivo hemostatic activity [133]. Moreover, the activation of cryopreserved platelets seems to occur via exposure of *P*-selectin at the platelet and microparticle membranes [120]. Measurement of dynamic light scattering (DLS) scores using the ThromboLUX^TM^ system showed significant differences in particle composition between RT-stored platelets and cold-stored or cryopreserved platelets, suggesting increased microparticulation in response to storage at low temperature [121].

Redox regulation of platelet microparticulation is still poorly understood, but elevated levels of microparticles are associated with inflammatory diseases, such as arterial thrombosis, diabetes, rheumatoid arthritis and ischemic events [134,135]. Holbrook et al. showed that thrombin stimulation of platelets induces the shedding of microparticles containing PDI and ERp57, both of which are disulfide isomerases [136]. We mentioned above that the PDI on the platelet surface can regulate the cysteine-rich β3 region of the αIIbβ3 integrin as well as the affinity of αIIbβ3 for fibrinogen. Essex and Li significantly downregulated the affinity of αIIbβ3 for PAC-1, specifically by inhibiting PDI activity with rabbit anti-PDI IgG [137]. Therefore, cold stored and cryopreserved platelets most likely show hyperactivation relative to RT-stored platelets due to increased microparticulation [120]. Nevertheless, PDI is not the only disulfide isomerase to act at the platelet surface. Holbrook and coworkers identified several new disulfide isomerases on the platelet surface using immunoblotting [136]. Confirming intense oxidoreductase activity at the platelet membrane, PDI was found to be released by platelets during injury as an activator of the tissue factor that initiates fibrin polymerization after thrombin activation [138]. Indeed, PDI catalyzes the formation of an intra-disulfide bond between a mixed disulfide (i.e., GSSR) and a free thiol on the tissue factor. Moreover, low temperature storage seems to affect redox regulation by enhancing the capacity of platelets to synthetize TxA_2_ via AA stimulation [139]. In spite of these differences relative to PI-treated platelets, a number of clinical studies have been conducted using platelets that were stored at low temperatures, and they were shown to be safe and thus to represent a real option for patients.

### 6.3. Whole Blood Inactivation and Cold Storage

Pathogen inactivation to prevent transfusion-transmitted infections has become a central paradigm in transfusion medicine. While several countries have adopted regulations for PIT use in PC manufacturing, others still need to upgrade their blood supply systems. PITs have been used during infection crises, for example in the Chikungunya outbreak on Reunion Island, to avoid the collapse of the blood component supply [140]. Therefore, PITs are currently part of the arsenal used to fight emerging pathogens. Unfortunately, UVA-based PITs cannot be used to inactivate red blood cell (RBC) concentrates or even whole blood. Due to the overlapping absorption spectra of hemoglobin and its high concentration in blood, technologies that employ UVA radiation damage RBCs, increasing the risk of an immune response in the recipient [141]. To overcome this barrier to use, a chemical-based S-303 inactivating system has been developed that uses glutathione combined with the amphipathic chemical compound S-303 [142]. The membrane-permeable compound comprises a linker that can covalently bind to RNA/DNA, blocking their replication, plus an inert tail reporter released upon the binding of nucleotides. Glutathione molecules, which are not membrane permeable, quench the S-303 side reactions, protecting proteins and other extracellular components. This system avoids the use of photochemistry to inactivate pathogens. The first evidence that S-303 can be used to treat whole blood was presented by Mufti et al. [143]. Treatment of whole blood could simplify the pathogen inactivation process, allowing the treated blood to subsequently be used to obtain plasma, platelets and RBC components via the buffy coat method. However, S-303-based inactivation does have an impact on cells. Indeed, erythrocyte concentrates showed decreased glycolysis and increased extracellular potassium levels after treatment with S-303 [143]. Due to its affordability and potential for widespread use, the idea of treating whole blood has also been tested using the Mirasol^®^ system. To assess the suitability of the UVB/riboflavin treatment for whole blood, the viability of the different components was tested in vitro. Schubert et al. found significant deterioration in plasma coagulation factor activation and increased RBC aging in components derived from whole blood that was PI-treated [144]. Interestingly, platelets showed fewer lesions than PI-treated PCs. Again, further studies are needed to clarify the effects of this system on specific molecular components.

As a final remark, it is important to notice that all developments and innovations in transfusion medicine must be based not only on scientific data but also on safety and economic issues.

## 7. Conclusions

In platelets, the ROS balance controls both platelet activation and the platelet aggregation process. In the context of transfusion medicine, PITs slightly disturb the resting state of platelets. The clinical impact of this disturbance is not completely understood, although the safety of PITs has been shown. However, in vitro tests show evidence of lesions in treated PCs. Considering the increase in the protein carbonylation level in PI-treated PCs, it is reasonable to suspect that these lesions activate platelets via ROS. ROS are very reactive small molecules that can transduce stimuli into biological responses. Although the link between second messenger ROS and platelet activation is now well known, the molecular mechanisms that regulate this process remain to be elucidated. As described here, the redox potential of cysteine plays a central role in signal transduction. Accordingly, PITs that produce oxidative stress could drive the creation of PCs that contain platelets that are slightly hyperactive, show increased lesions with increased storage time, and may not be well suited to the characteristics of the recipient. Alternative storage conditions could be suited to other clinical status; we must more fully understand the in vitro functionality of platelets to fully understand the clinical implications of PITs.

Advanced proteomics, and in particular redox proteomics, are of primary importance for deciphering the defined steps in the platelet activation and aggregation processes. As highlighted here, the identification of platelet sulfenylation sites, among other oxPTM sites, will help researchers select candidate proteins that are sensitive to ROS and help to better characterize the final labile blood product. The discovery of these oxidative mechanisms could go beyond concerns about transfusion and give us a better understanding of inflammatory diseases that are accompanied by platelet hyperactivity [127].

Finally, from the viewpoint of blood centers, further basic and “omic” research that investigates redox regulations, as reviewed here, will be helpful for developing innovative and improved strategies for preparing and storing platelets. Although this work is promising, the relationships between platelet biochemistry and clinical effects must be elucidated further, and redox proteomics studies of PCs should ideally be accompanied by functional tests. Moreover, it is important to question the value and potential of current PITs and to think about the possibility of novel inactivation systems that do not rely on oxidative stress, such as whole blood inactivation, which does not use UV light.

## Figures and Tables

**Table 1:** Redox cysteine oxidation states.

Name	Formulae	Oxidation State
Cysteine	R–SH	−II
Disulfide bridge	R–S–S–R	−I
Glutathionylation	R–S–S–G	−I
Sulfenic acid	R–SOH	0
Sulfinic acid	R–SO_2_H	+II
Sulfonic acid	R–SO_3_H	+IV

## Abbreviations

2D-GE	Two dimensional-gel electrophoresis
F-actin	Actin filaments
AA	Ascorbic acid
CCI	Corrected count increment
COX1	Cyclooxygenase 1
CyPA	Cyclophilin A
DCF	2′,7′-dichlorofluorescein
F2-IsoP	F2 isoprostane
GP-Ib-IX	Glycoprotein Ib-IX
GPVI	Glycoprotein VI
H_2_DCFDA	2′,7′-dichlorodihydrofluorescein diacetate
IP3	Isoprostane 3
LAT	Linker of activated T-cells
mPT	Mitochondria permeability transition
NOX	NADPH oxidase
PC	Platelet concentrate
PDI	Protein disulfide isomerase
PGG2	Prostaglandin G2
PI	Pathogen inactivation
PIT	Pathogen inactivation technology
PLA_2_	Phospholipase A2
PLCg-2	Phospholipase Cg 2
PrxII	Peroxiredoxin-II
PTM	Post-translational modification
oxPTM	Oxidative PTM
RBC	Red blood cell
ROS	Reactive oxygen species
RT	Room temperature
SOD	Superoxide dismutase
TRAF4	TNF receptor associated factor 4
TXA2	Thromboxane A2
